# *HOTAIR/miR-203/CAV1* Crosstalk Influences Proliferation, Migration, and Invasion in the Breast Cancer Cell

**DOI:** 10.3390/ijms231911755

**Published:** 2022-10-04

**Authors:** Fuxiu Shi, Xinyue Chen, Yi Wang, Yujie Xie, Junpei Zhong, Kangtai Su, Miao Li, Yuqiu Li, Qing Lin, Youjia Zhou, Jie Wang, Lixia Xiong

**Affiliations:** 1 Department of Pathophysiology, Medical College, Nanchang University, 461 Bayi Road, Nanchang 330006, China; 2 Second Clinical Medical College, Nanchang University, Nanchang 330006, China; 3 College of Pharmacy, Nanchang University, Nanchang 330006, China; 4 First Clinical Medical College, Nanchang University, Nanchang 330006, China; 5 Queen Mary School, Nanchang University, Nanchang 330006, China; 6 Key Laboratory of Functional and Clinical Translational Medicine, Xiamen Medical College, Fujian Province University, Xiamen 361023, China

**Keywords:** breast cancer, HOTAIR, miR-203, CAV1, ceRNA, invasion, migration

## Abstract

In recent years, malignant breast cancer metastasis has caused a great increase in mortality. Research on the genetic and molecular mechanisms of malignant breast cancer has continued to deepen, and targeted therapy has become the general trend. Among them, competing endogenous RNA (ceRNA)-related molecules have received much attention. Homeobox transcript antisense RNA *(HOTAIR*) has been reported to function extensively as a ceRNA in breast cancer. Notably, *miR-203* and *Caveolin 1* (*CAV1)* have also been found to play a role in breast cancer. However, the relationship between the three remains unclear. In this study, we present a new mechanic through bioinformatics tool and basic experiments: the *HOTAIR*/*miR-203*/*CAV1* axis, which complemented the role network of *HOTAIR* as a ceRNA, thus, it will provide a novel potential idea for breast cancer research and therapy.

## 1. Introduction

According to the latest statistics released by the International Agency for Research on Cancer (IARC) in December 2020, breast cancer accounted for 11.7% of new tumor cases in 2020, surpassing lung cancer, which accounted for 11.4% [1]. Breast cancer has become the most common malignant tumor type in the world. Therefore, the prevention, diagnosis, and treatment of breast cancer are urgent. The significance of analyzing breast cancer’s metastasis mechanism and studying the mechanism of malignant proliferation, invasion, and migration of breast cancer cells has reached its peak. Homeobox (HOX) transcript antisense RNA (*HOTAIR*) was first discovered accidentally in 2007 and is located at 12q13.13, between *HOXC11* and *HOXC12* on chromosome 12 [2]. Therefore, it is also called intergenic long non-coding RNA (lncRNA), which contains 2158 nucleotides and 6 exons. *HOTAIR* can regulate gene expression epigenetically in a manner similar to scaffolding and can also interfere with the expression of signaling molecules associated with breast cancer development in breast cancer. Qian L. et al. found that up-regulated *HOTAIR* in breast tissue can enhance the expression of the DNA damage repair factors and regulate cell cycle and apoptosis [3]. The overexpression of *HOTAIR* can activate estrogen receptor transcription, inducing breast cancer drug-resistant and promoting cancer cell proliferation [4]. A recent study analyzed the in situ expression of *HOTAIR* in breast cancer patients and showed that a high expression of HOTAIR in tumor tissue is closely related to lymph node metastasis [5]. A population-based case-control study found that *HOTAIR* is associated with breast cancer risk and prognosis [6]. Based on the important role of *HOTAIR* in breast cancer progression, a recent review summarized the potentially promising therapeutic function of *HOTAIR* in breast cancer [7]. However, the underlying mechanism of *HOTAIR* in breast cancer requires more research.

*Caveolin 1* (*CAV1*) is the gene encoding for caveolin-1. The expression of the *CAV1* gene is associated with many human diseases, including pulmonary hypertension, hypertriglyceridemia, and cancer. It has been reported that *CAV1* plays a key role in breast cancer cell proliferation, apoptosis, autophagy, invasion, migration, and breast cancer metastasis [8,9,10,11,12]. Another study confirmed the absence of *CAV1* gene mutations in human breast cancer [13]. It is only that the expression level of *CAV1* is regulated by other factors, and it plays a dual role in inhibition or promotion in the process of breast cancer [14]. In the study of mechanisms in breast cancer, *CAV1* has been our target, and we strive to identify the key molecules that act on it.

It has been reported that lncRNAs regulate mRNA expression by binding specific microRNAs (miRNAs) as competing endogenous RNAs (ceRNAs) [15]. Therefore, the physical interaction of ceRNA-miRNAs leads to a complex regulatory network of ceRNA/miRNA/mRNA that controls gene expression at the transcriptional, post-transcriptional, and post-translational levels [16,17]. Some studies have found that these interacting ceRNA/miRNA/mRNA networks are involved in many cellular processes and human diseases, including tumorigenesis [18,19,20]. The ceRNA/mRNA network has also been reported in the study of breast cancer mechanisms. Using a weighted gene co-expression network analysis (WGCNA) algorithm, they found a ceRNA- lncRNA *TRPM2* that promotes the proliferation of BRCA cells and inhibits apoptosis through the *TRPM2-AS*/*miR-140-3p*/*PYCR1* axis [21]. This further inspires us to explore the miRNA that can connect lncRNA*HOTAIR* and *CAV1* and improve the role of the ceRNA/mRNA network in the progression of breast cancer.

In this study, we first analyzed the differential expression of *HOTAIR* in breast cancer and normal tissues by using database and online messaging tools, screened the possible *HOTAIR*-binding miRNA, enriched some of the miRNAs, verified the role of *miR-203* in cancer, and chose the *miR-203* which we are interested in. Then, we analyzed the key protein molecules and their corresponding genes by protein–protein interaction (PPI) and found that *CAV1* was included in the PPI network. The dual-luciferase reporter gene, cell proliferation, migration, and invasion in vitro were used to verify the sponge effect of *HOTAIR* on *miR-203* and the regulation of *miR-203* on *CAV1* expression. Finally, the role of the *HOTAIR*/*miR-203*/*CAV1* axis in the proliferation, invasion, and migration of the breast cancer cell line MDA-MB-231 was clarified.

## 2. Results

### 2.1. High Expression of HOTAIR in Breast Cancer

**Figure 1:**
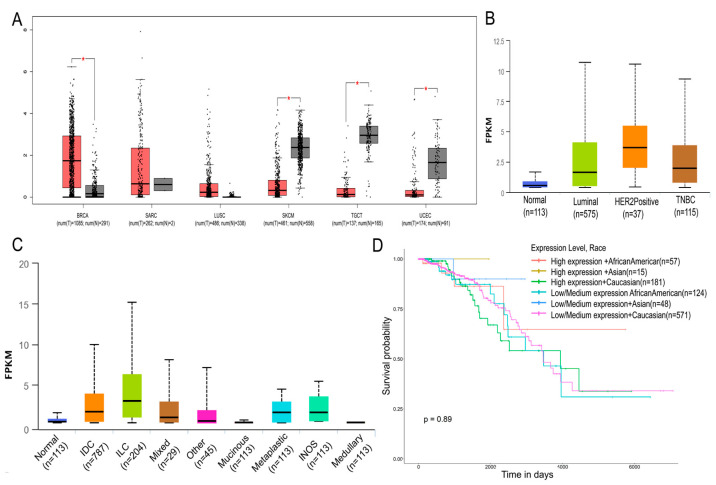
*HOTAIR* is highly expressed in breast cancer. Data analysis from GEPIA and UALCAN. (**A**) Among various cancers, *HOTAIR* was significantly overexpressed in breast cancer. (**B**) *HOTAIR* was significantly overexpressed in luminal breast cancer, *HER2*-positive breast cancer, and TNBC. (**C**) Compared with normal tissues, *HOTAIR* was significantly overexpressed in breast invasive carcinoma. (**D**) Breast cancer patients with high expression of *HOTAIR* might have a poorer prognosis. BRCA, breast invasive carcinoma; LUSC, lung squamous cell carcinoma; SARC, sarcoma; SKCM, skin cutaneous melanoma; TGCT, testicular germ cell tumors; TNBC, triple-negative breast cancer; UCEC, uterine corpus endometrial carcinoma; IDC, invasive ductal carcinoma; ILC, invasive lobular carcinoma; TNBC, triple-negative breast cancer. Values were significantly different compared with the corresponding control value at * *p* < 0.05.

To reveal the function of *HOTAIR* in cancer, we first used GEPIA (http://gepia.cancer-pku.cn/detail.php, accessed on 12 March 2022) to analyze the expression of *HOTAIR* in different cancers. The results showed that, compared with normal tissues, *HOTAIR* was highly expressed in breast cancer (Figure 1A). Through the UALCAN (http://ualcan.path.uab.edu/index.html, accessed on 12 March 2022) online tool to analyze the TCGA database, the results showed that *HOTAIR* was significantly overexpressed in luminal breast cancer, *HER2*-positive breast cancer, and triple-negative breast cancer (TNBC) (Figure 1B). Based on the subclasses, the UALCAN results showed that *HOTAIR* was overexpressed in breast invasive carcinoma compared with normal tissues (Figure 1C). The Kaplan–Meier curves and log-rank analysis results suggested that patients with a high expression of *HOTAIR* may have a worse prognosis (Figure 1D).

### 2.2. Up-Regulation of HOTAIR Promotes the Proliferation, Invasion, and Migration of MDA-MB-231 Cells

**Figure 2:**
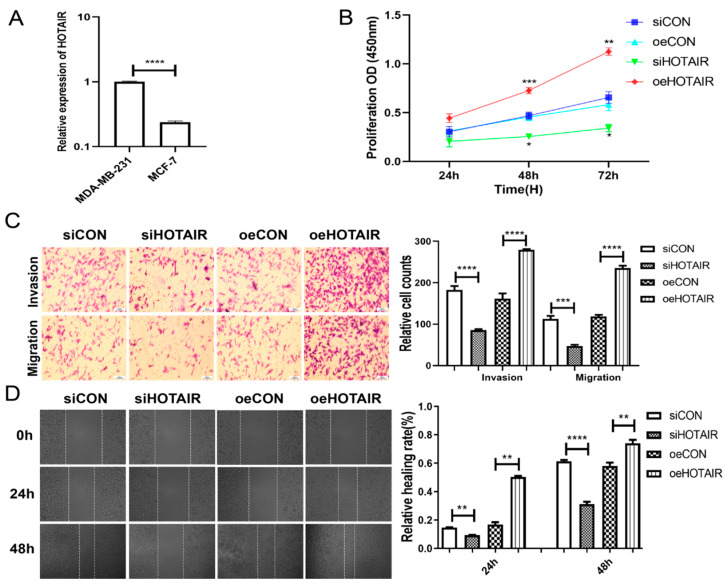
Up-regulation of *HOTAIR* promotes the proliferation, invasion, and migration of MDA-MB-231 cells. (**A**) RT-qPCR results showed that the expression of *HOTAIR* was higher in MDA-MB-231 than in MCF-7. (**B**) CCK-8 results showed that the proliferation ability of MDA-MB-231 cells in the si*HOTAIR* group decreased after 48 h and 72 h compared to the siCON group. Compared with the oeCON group, the proliferation ability of MDA-MB-231 cells in the oe*HOTAIR* group was increased. (**C**) Transwell assay showed that after 48 h, the invasion and migration number of MDA-MB-231 cells in the *HOTAIR* overexpression group was significantly higher, while that in the si*HOTAIR* group was lower than that in the NC group. (**D**) The results of the wound healing assay showed that compared with the NC group, the migration ability of MDA-MB-231 cells in the si*HOTAIR* group was significantly decreased, and the migration ability of the cells in the overexpression *HOTAIR* group was significantly higher than that in the NC group. Values were significantly different compared with the corresponding control value at * *p* < 0.05, ** *p* < 0.01, *** *p* < 0.001, **** *p* < 0.0001.

The expression of *HOTAIR* was higher in MDA-MB-231 than in MCF-7 (Figure 2A); thus, we selected a highly invasive breast cancer cell line, MDA-MB-231 (a type of TNBCcells), for cell transfection in the subsequent experiments. The results of CCK-8 showed that the proliferation rate of down-regulated *HOTAIR* cells decreased, while the proliferation rate of overexpressed *HOTAIR* cells increased significantly (Figure 2B). The transwell assay results showed that the ability of migration and invasion in overexpressed *HOTAIR* cells was significantly increased, while in down-regulated *HOTAIR* cells, it was decreased (Figure 2C). The wound healing assay results revealed that *HOTAIR* could strikingly enhance the migration of MDA-MB-231 (Figure 2D). These results all proved that the up-regulation of *HOTAIR* could promote the proliferation, migration, and invasion of MDA-MB-231 cells.

### 2.3. HOTAIR Binds to miR-203 and Acts as a Molecular Sponge for miR-203

**Figure 3:**
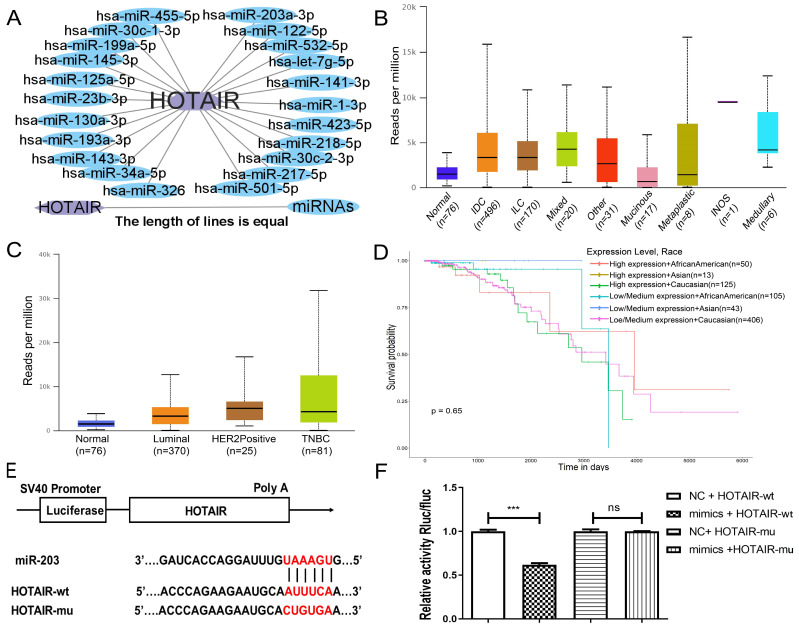
*miR-203* functions in cancer, and *HOTAIR* acts as a molecular sponge for *miR-203*. (**A**) Data of possible *HOTAIR*-bound miRNAs were derived by comprehensive analyses of ENCORI and DAVID. The HOTAIR-miRNA network map was made by Cytoscape3.0 software. (**B**) Compared with normal tissues, *miR-203* was overexpressed in breast invasive carcinoma through the UALCAN analysis of the TCGA database. (**C**) *miR-203* was significantly overexpressed in luminal breast cancer, *HER2*-positive breast cancer, and TNBC. (**D**) Breast cancer patients with high expression of *miR-203* might have a poorer prognosis. (**E**) Putative complementary sites between *HOTAIR* and *miR-203*. Mutations were generated in the *HOTAIR* nucleotides complementary to *miR-203*. (**F**) Fluorescence expression was detected by the dual-luciferase reporter gene. Compared with the NC group, the luciferase activity of the *miR-203* mimics/*HOTAIR*-wt group decreased, while the luciferase activity of the *miR-203* mimics/*HOTAIR*-mu group did not change significantly (*p* > 0.05). Values were significantly different compared with the corresponding control value at *** *p* < 0.001. ns > 0.05. K means 1000. IDC, invasive ductal carcinoma; ILC, invasive lobular carcinoma; TNBC, triple-negative breast cancer.

To study the relation between *HOTAIR* (as ceRNA) and *miR-203* in breast cancer, we predicted the potential miRNAs targets of *HOTAIR* by using ENCORI (StarBase, https://starbase.sysu.edu.cn/index.php, accessed on 12 March 2022) and DAVID (https://david.ncifcrf.gov/tools.jsp, accessed on 12 March 2022). We exported the data (Table S1) from the two databases. Software Cytoscape 3.0 was used to construct a network map of miRNAs that may bind to *HOTAIR* (Figure 3A). Of note, *miR-203* was one of the targets (Figure 3A). Then, we performed GO functional annotation and pathway enrichment analysis using miEAA 2.0 (https://ccb-compute2.cs.uni-saarland.de/mieaa, accessed on 12 March 2022) to examine the functions of these miRNAs. The GO analysis was conducted at three sublevels: biological process (BP), molecular function (MF) and molecular composition (CC). The results of MF and CC are the same. The results showed that the overexpression of *miR-203* was significantly enriched in the regulation of angiogenesis, cell proliferation, and apoptosis, such as angiogenesis, blood vessel morphogenesis, and the positive regulation of the cell population in the BP category (Figure S1); negative regulation of anoikis, wound healing, B-cell lymphoma-2 family protein complex, and the extrinsic apoptotic signaling pathway in the MF and CC categories (Figures S2 and S3). KEGG’s cell signaling pathway was also conducted. The *miR-203*-enriched KEGG pathways were MicroRNAs in cancer, pathways in cancer and Adherens junction (Figure S4). Compared with normal tissues, *miR-203* was overexpressed in breast invasive carcinoma through the UALCAN analysis of the TCGA database (Figure 3B). Moreover, *miR-203* was significantly overexpressed in luminal breast cancer, HER2-positive breast cancer, and TNBC (Figure 3C). The Kaplan–Meier analysis suggested that patients with a high expression of *miR-203* may have a worse prognosis (Figure 3D). From the above analysis, it can be concluded that *miR-203* plays an important role in the proliferation, survival, migration, and invasion of cells, especially cancer cells. Notably, these roles of *miR-203* are similar to *HOTAIR*. Therefore, we have reason to believe that *HOTAIR* is one of the ceRNAs of *miR-203*. To further verify the binding of *HOTAIR-miR-203* in cancer cells, we constructed the wild-type plasmid *HOTAIR*-wt of *HOTAIR* that contains the complementary sequence of *miR-203*, and the mutant plasmid *HOTAIR*-mu with the mutation of the complementary binding sequence (Figure 3E). Compared with the NC group, the luciferase activity of the *miR-203* mimics + *HOTAIR*-wt group was decreased, while the *miR-203* mimics + *HOTAIR*-mu group luciferase activity changes were less pronounced (Figure 3F), showing the existence of binding between *HOTAIR* and *miR-203*.

### 2.4. HOTAIR Reverses the Effect of miR-203 on MDA-MB-231 Cells in Proliferation, Invasion, and Migration

**Figure 4:**
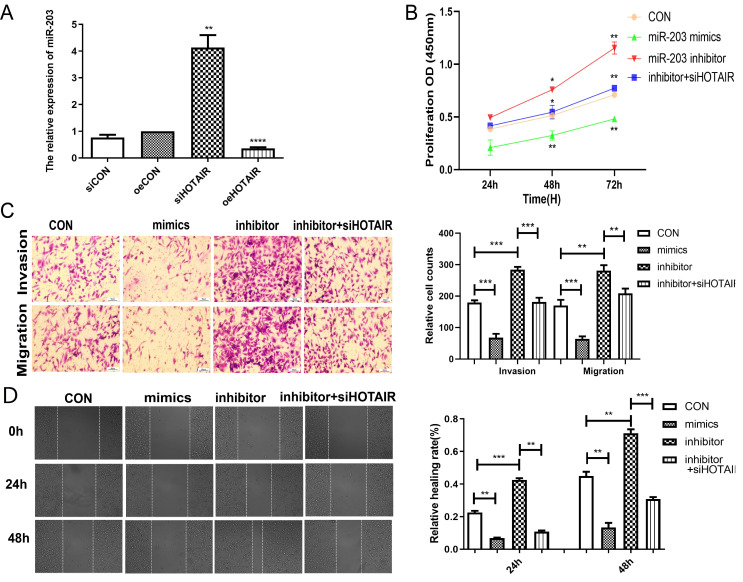
*HOTAIR* reverses the effect of *miR-203* on MDA-MB-231 cells in proliferation, invasion, and migration. (**A**) RT-qPCR results showed that compared with the NC group, the expression level of *miR-203* was significantly increased after down-regulation of *HOTAIR*, while the expression level of *miR-203* was extremely significantly decreased after up-regulation of *HOTAIR*. (**B**) CCK-8 results showed that after 48 h and 72 h, compared with the NC group, the cell proliferation rate in the *miR-203* inhibitor group was significantly higher than that in the *miR-203* mimics group; compared with the *miR-203* inhibitor group, the rate of cell proliferation decreased in the inhibitor + si*HOTAIR* group. (**C**) The transwell invasion and migration assay results showed that, compared with the NC group, the number of cell invasions and migrations in the *miR-203* inhibitor group was significantly increased. In the inhibitor + si*HOTAIR* group, the number of cell invasions and migrations significantly decreased. (**D**) The results of the wound healing assay showed that the migration area in *miR-203* inhibitor cells was significantly increased, while in the inhibitor + si*HOTAIR* group, the migration ability of cells was decreased. Values were significantly different compared with the corresponding control value at * *p* < 0.05, ** *p* < 0.01, *** *p* < 0.001, **** *p* < 0.0001.

To further verify the role of *HOTAIR/miR-203* in breast cancer progression, we transfected the MDA-MB-231 cells with the *HOTAIR* plasmid. The RT-qPCR results showed that the expression level of *miR-203* was significantly increased when *HOTAIR* was down-regulated, and the expression level of *miR-203* was significantly decreased when *HOTAIR* was up-regulated (Figure 4A). The results of the CCK assay showed that inhibiting the expression of *miR-203* would promote the proliferation of MDA-MB-231 cells, and down-regulating *HOTAIR* reversed the effect of the *miR-203* inhibitor on the proliferation of MDA-MB-231 cells (Figure 4B). The transwell assays showed that the inhibition of *miR-203* expression promoted the migration and invasion of MDA-MB-231 cells, and the down-regulation of *HOTAIR* reversed the effect of the *miR-203* inhibitor on the promotion of MDA-MB-231 cell migration and invasion (Figure 4C). The wound healing assay results showed that inhibiting the expression of *miR-203* would promote the migration of MDA-MB-231 cells, and down-regulating *HOTAIR* reversed the effect of the *miR-203* inhibitor on promoting the migration of MDA-MB-231 cells (Figure 4D). The above results all proved that *HOTAIR/miR-203* could regulate the proliferation, migration, and invasion of MDA-MB-231 cells.

### 2.5. miR-203 Binds to CAV1 and Inhibits the Expression of CAV1

**Figure 5:**
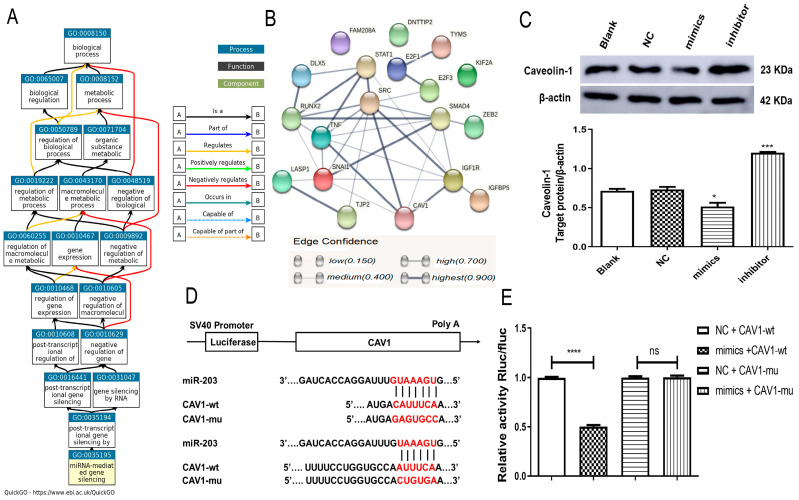
*miR-203* binds to *CAV1* and inhibits the expression of *CAV1*. (**A**) Gene Ontology (GO-Biological Process) for *miR-203* involved in gene silencing (GO: 0035195). (**B**) PPI network diagram between *miR-203* target gene-encoded proteins and *CAV1*-encoded proteins derived from STRING. (**C**) Western blot detection showed that the expression level of caveolin-1 was significantly increased in the *miR-203* down-regulated group. (**D**) Putative complementary sites between *miR-203* and *CAV1*. Mutations were generated in the *CAV1* nucleotides complementary to *miR-203*. (**E**) Dual-luciferase reporter gene detection of the interaction between *miR-203* and *CAV1*. Compared with NC, the expression of luciferase in mimics + *CAV1*-wt decreased, and the expression of luciferase in mimics + *CAV1*-mu was unchanged. Values were significantly different compared with the corresponding control value at * *p* < 0.05, *** *p* < 0.001, and **** *p* < 0.0001. ns > 0.05.

The last link is missing in our constructed ceRNA/miRNA/mRNA axis. Therefore, GO (GO-Biological Process) in GeneCards (https://www.genecards.org/, accessed on 12 March 2022) for the *miR-203* involved in gene silencing by miRNA was analyzed (GO:0035195, Figure 5A). In addition, we used the online tool, miEAA 2.0, to enrich the target genes corresponding to the miRNAs (including *miR-203*) that may be bound by *HOTAIR*, and screened all the target genes containing *miR-203* in the enrichment (Table S2). Then, STRING (https://cn.string-db.org/, accessed on 12 March 2022) was used to make a network map of the interaction (PPI) between the protein encoded by the target gene and the protein encoded by *CAV1* (Figure 5B). Notably, *CAV1* connected five edges as one of the nodes (Figure 5B), indicating that *CAV1* may be in the regulatory network of *miR-203.* Therefore, we transfected the MDA-MB-231 cells with *miR-203* mimics and *miR-203* inhibitors to verify the effect of *miR-203* on *CAV1* expression. The Western blot results showed that when the expression of *miR-203* was down-regulated, the expression level of caveolin-1 was significantly increased. When the expression of m*iR-203* was up-regulated, the expression level of caveolin-1 was decreased (Figure 5C). To further verify whether *miR-203* binds to *CAV1*, we constructed the wild-type plasmid *CAV1*-wt that contains the complementary sequence of *miR-203*, and mutant plasmid *CAV1*-mu, with a mutation of the complementary binding sequence (Figure 5D). The experimental results obtained by the dual-luciferase reporter gene assay are shown in Figure 5E. Compared with the NC group, transfection *miR-203* could significantly down-regulate the luciferase activity of the CAV1-wt group. However, transfection of *miR-203* could not significantly change the luciferase activity of the *CAV1*-mu group, which proved that *miR-203* could directly bind to *CAV1*. Therefore, *miR-203* could interact with *CAV1*, and the overexpression of *miR-203* could inhibit the expression of *CAV1*.

### 2.6. miR-203 Restrains the Function of CAV1 on MDA-MB-231 Cells in Proliferation, Invasion, and Migration

**Figure 6:**
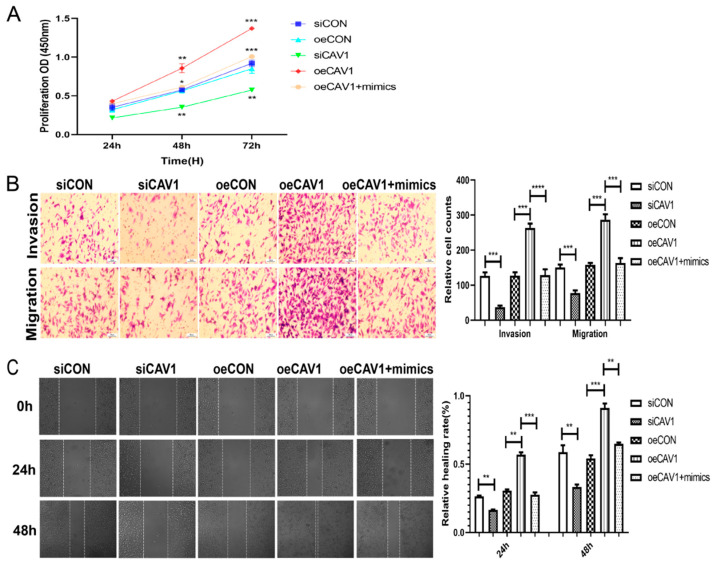
*miR-203* inhibits the effect of *CAV1* on MDA-MB-231 cells in proliferation, invasion, and migration. (**A**) CCK-8 results showed that the down-regulation of *CAV1* significantly reduced the cell proliferation rate after 48 h and 72 h. Compared with the oe*CAV1* group, the proliferation rate of the cells in the co-transfected m*iR-203* (oe*CAV1* + mimics) group decreased. (**B**) The transwell invasion and migration assay results showed that the number of MDA-MB-231 cells invading and migrating in the oe*CAV1* group increased. The number of cell invasions and migrations in the oe*CAV1* + mimics group was lower than that in the oe*CAV1* group. (**C**) The wound healing assay showed that the migration area of cells in the oe*CAV1* group increased. The migration area of cells in the oe*CAV1* + mimics group was smaller than that in the oe*CAV1* group. Values were significantly different compared with the corresponding control value at * *p* < 0.05, ** *p* < 0.01, *** *p* < 0.001, **** *p* < 0.0001.

We further investigated, in vitro, that *miR-203/CAV1* regulated the proliferation, invasion, and migration of MDA-MB-231 cells. The results of CCK-8 showed that the overexpression of *CAV1* could promote the proliferation of MDA-MB-231 cells, and up-regulation of the expression of *miR-203* could reverse the promoting effect of the overexpression of *CAV1* on the proliferation rate of MDA-MB-231 cells (Figure 6A). The transwell assay showed that the overexpression of *CAV1* could promote the migration and invasion of MDA-MB-231 cells, and up-regulation of *miR-203* reversed the promoting effect of *CAV1* overexpression on the migration and invasion ability of MDA-MB-231 cells (Figure 6B). The wound healing assay showed that the overexpression of *CAV1* could promote the migration of MDA-MB-231 cells, and up-regulation of *miR-203* reversed the promotion of *CAV1* overexpression on the migration ability of MDA-MB-231 cells (Figure 6C). The above in vitro experiments all proved that the overexpression of *CAV1* could promote the progression of breast cancer cells, and the interaction of *miR-203/CAV1* could regulate the proliferation, invasion, and migration of MDA-MB-231 cells.

## 3. Discussion

LncRNA is a type of non-coding RNA, exerting as ceRNA. In recent years, the role of the ceRNA networks in cancer has been extensively studied, linking the functions of protein-coding mRNAs to those of non-coding RNAs [17]. miRNAs are also non-coding RNAs with a length of about 22 nt and play a crucial role in regulating gene expression [22]. They exert regulatory effects by binding to the 3′-untranslated region of target mRNAs, leading to mRNA degradation or silencing in a sequence-specific manner [22]. Given that any transcript containing miRNA-response elements could theoretically function as a ceRNA, they likely represent a broad form of post-transcriptional regulation of gene expression in physiology and pathology. *HOTAIR* is the first lncRNA discovered to have trans-regulatory roles [2]. Since the discovery of *HOTAIR*, multiple studies have elucidated its critical role in tumor growth, apoptosis, invasion, metastasis, tumor stem cell differentiation, and drug resistance. Notably, *HOTAIR* has been reported to function extensively as a ceRNA in breast cancer. Zhao W. et al. found that *HOTAIR* affected MDA-MB-231 cell growth and apoptosis through the *miR-20a-5p*/*HMGA2* pathway [23]. Wu D. et al. found that the *HOTAIR*/*miR-129-5p*/*FZD7* axis could accelerate breast cancer progression [24]. In addition, *HOTAIR* could increase radioresistance in breast cancer by promoting *HSPA1A* expression through binding to *miR-449b-5p* in MDA-MB-231 cells [25]. 

Breast cancer studies are limited to a few cell lines, with MCF-7, T47D, and MDA-MB-231 accounting for the majority of studies [26]. Notably, MDA-MB-231 is a highly aggressive and poorly differentiated triple-negative breast cancer cell line [27]. In recent years, articles on tumor metastasis, drug resistance, and treatment based on MDA-MB-231 have been emerging. Research on *HOTAIR* has been studied in MDA-MB-231. It is of interest to continue to study MDA-MB-231 to construct a more complete network of actions of *HOTAIR*. Additionally, in our study, we found that the level of *HOTAIR* was higher in MDA-MB-231 than in MCF-7 (Figure 2A). In addition, the results (Figure 1C) showed that *HOTAIR* was higher in TNBC, as known to all, that MDA-MB-231 is a type of TNBC cell. Therefore, we chose the MDA-MB-231 cell line. In this study, we demonstrated that the overexpression of *HOTAIR* significantly promoted the proliferation, invasion, and migration in MDA-MB-231 cells, which was consistent with the predictions using bioinformatics analysis andprevious research.

Further, *miR-203*, a member of the miRNA family, has been confirmed to be involved in regulating the proliferation, differentiation, metastasis, invasion, and apoptosis of tumor cells [28,29]. Currently, only three studies have addressed the role of *miR-203*′s link with *HOTAIR* in cancer [30,31,32]. The connection between the two deserves further investigation. In this study, we predicted the role of *miR-203* in biological and cancer processes through database information. Notably, *HOTAIR* can compete for binding to *miR-203*, which is a molecular sponge of *miR-203*. The dual-luciferase reporter gene assay showed the combination of the two. Furthermore, we demonstrated that the expression of *miR-203* was significantly decreased after the overexpression of *HOTAIR*. The inhibition of *HOTAIR* reversed the promotion of breast cancer cell progression induced by *miR-203* down-expression. That is, *HOTAIR* could reverse the inhibitory effect of *miR-203* on the proliferation, invasion, and migration of MDA-MB-231 cells. Notably, through the TCGA database, we analyzed the expression of *miR-203* in breast cancer and found that the expression of *miR-203* was different in different breast subtypes. However, its expression was higher than that in normal tissues. In addition to our results, other existing experimental data showed that miRNA was used as a tumor suppressor, such as *miR-203*, identified as a transcript of matrix stiffness inhibition, which was negatively correlated with the malignant proliferation of breast epithelium [33]. Further, *miR-203* also inhibited the migration ability of MDA-MB-231 cells by targeting *PRKCQ* [34]. There are also studies that directly conclude that LncRNA *DLG1-AS1* may promote cancer cell proliferation in TNBC by down-regulating the tumor suppressor, *miR-203* [35]. This suggests that the role of *miR-203* in breast cancer is intriguing, and the mechanism behind its expression, regulation by other molecules, and tumor inhibition is worthy of further exploration.

Recently, *CAV1* has been found to be actively involved in human tumor progression [36]. On the one hand, high expression of *CAV1* has been reported to drive tumorigenesis by inhibiting apoptosis and promoting anchorage-independent growth, drug resistance, and metastasis [37,38,39]. For example, *CAV1* expression in pancreatic cancer cells was found to be positively correlated with cachectic states [40]. On the other hand, *CAV1* acts as a tumor suppressor in some cases, as its low expression favors tumor progression. For instance, Geletu et al. found that *CAV1* down-regulation accelerated the proliferation of lung cancer cells via the Cadherin-11/Stat3 axis [41]. Notably, in recent studies, the dual role of *CAV1* is evident in breast cancer progression. Loss of stromal *CAV1* in human breast cancer was associated with a poor clinical outcome [42]. *CAV1* could suppress breast cancer development [43]. However, Dong et al. found that a high expression of *CAV1* promoted the migration and invasion of breast cancer cells, supporting its oncogenic role [9]. The dual role of *CAV1* in breast cancer deserves further exploration. Moreover, the regulation of *CAV1* expression also remains poorly understood. Therefore, our team has been devoted to the related research of *CAV1*, trying to elucidate the mechanism of *CAV1* in breast cancer progression. Note, however, that the role of *CAV1* in association with *miR-203* in cancer has only been elucidated in renal cell carcinoma [44]. In this study, the down-regulation of *CAV1* expression attenuated the proliferation, invasion, and migration of the breast cancer cell MDA-MB-231. We used bioinformatics technology to find the *miR-203* molecule that can affect the expression of *CAV1*. The binding of *CAV1* and *miR-203* was further verified by basic experiments. Moreover, the overexpression of *miR-203* significantly down-regulated *CAV1*’s expression and reversed the promotion effect of the MDA-MB-231 cells’ progression induced by *CAV1*.

## 4. Materials and Methods

### 4.1. Database Search

Gene Expression Profiling Interactive Analysis (GEPIA; http://gepia.cancer-pku.cn/detail.php, accessed on 12 March 2022) [45] was used to analyze the expression of HOTAIR in invasive breast cancer. UALCAN (http://ualcan.path.uab.edu/index.html, accessed on 12 March 2022) was used to analyze the differential expression of *HOTAIR* and *miR-203* in breast cancer subtypes and survival analysis in the TCGA database [46]. The ENCORI (StarBase, https://starbase.sysu.edu.cn/index.php, accessed on 12 March 2022) database [47] and DAVID (https://david.ncifcrf.gov/tools.jsp, accessed on 12 March 2022) database [48] were used to analyze the miRNAs interacting with *HOTAIR*. Cytoscape (3.9.0) was used to draw the HOTAIR-miRNA network map and select the key molecules on the network map. Gene Ontology/Kyoto Encyclopedia of Genes and Genomes (GO/KEGG) analysis on the miRNA set was performed by using the miRNA enrichment analysis and annotation tool, miEAA 2.0, online (https://ccb-compute2.cs.uni-saarland.de/mieaa, accessed on 12 March 2022) [49], and GeneCards (https://www.genecards.org/, accessed on 12 March 2022). Then, enrich the related target genes of *miR-203*, and use the STRING (https://cn.string-db.org/, accessed on 12 March 2022) database [50] for the proteins encoded by the target genes to export the related protein–protein interaction (PPI) network. Finally, combined with the *CAV1*, we focused on and analyzed whether it has a role with the above proteins.

### 4.2. Cell Culture

The human breast cancer cell line (MDA-MB-231and MCF-7) was purchased from Shanghai Cell Bank, the Chinese Academy of Sciences. The human embryonic kidney cell line (HEK-293T) was provided by Shanghai Hanheng Biotechnology Co., Ltd., Shanghai, China. The cells were cultured in DMEM (high glucose) (Hyclone, Logan, UT, USA), supplemented with 10% fetal bovine serum (FBS) (BI, KibbutzBeit Haemek, Israel) and 1% penicillin-streptomycin (Solarbio, Beijing, China) in a cultured environment of 37 °C constant temperature, 5% CO_2_ incubator.

### 4.3. Cell Transfection

Before transfection, 3.5 × 10^5^ cells were seeded in each well in a six-well plate. When the cells grew to about 70% of the bottom of the culture dish, Lipofectamine 3000 (Thermo Fisher, 81 Wyman Street, Waltham, MA, USA) and Opti-MEM serum-free medium (Gibco, Grand Island, NY, USA) were used for *HOTAIR* overexpression plasmid (*HOTAIR* homo pcDNA3.1, oe*HOTAIR*). Further, siRNA (siRNA 536, si*HOTAIR*), miRNA mimics (*miR-203* mimics), miRNA inhibitor (*miR-203* inhibitor), *CAV1* overexpression plasmid (*CAV1* homo pcDNA3.1, oe*CAV1*), siRNA (siRNA710, si*CAV1*), negative control (NC, down-regulated NC is siCON, overexpressed NC is oeCON) were prepared as a transfection complex and added to six wells. The cells were transfected in the plate. The above pcDNA and siRNA were constructed by Suzhou Gene, and the cells were collected after 24 h to extract total RNA to determine the transfection efficiency by qPCR. The sequences are shown in Table S3.

### 4.4. RNA Extraction and Quantitative Real-Time PCR (RT-qPCR)

Total RNA was isolated from the cells 24 h post-transfection after RNA quantification according to the kit instructions (Omega, Madison, WI, USA), and the reaction system was prepared according to the instructions. RNA (2–5 ug per system) was reverse transcribed into cDNA by using the PerfectStartTM Green qPCR SuperMIX (TransGen Biotech, Beijing, China) and StepOne^TM^ qRT PCR machine (ABI, Waltham, MA, USA) real-time device under the following conditions: 42 °C for 30 min, 85 °C for 5 s. The qPCR system was prepared according to the instructions of the TransStart^®^ Tip Green qPCR SuperMix kit (TransGen Biotech, Beijing, China). The qPCR was performed under the following conditions: 94 °C for 30 s, 40 cycles of 94 °C for 5 s, and 62 °C for 30 s. All results were normalized to *U6* and glyceraldehyde-3-phosphate dehydrogenase (*GAPDH*). The relative expression level of miRNA was detected by the −2^∆∆Ct^ method. All primer pairs were synthesized by Sangon (Shanghai, China), and the sequences are shown in Table S4

### 4.5. Cell Proliferation Assay

The Cell Counting Kit-8 (CCK-8) (TransGen Biotech, Beijing, China) was used to detect the proliferation of MDA-MB-231, and the cells in the logarithmic growth phase that had been transfected in the early stage were used to inoculate 1000 cells per well in a 96-well plate. More than 4 duplicate wells were set in the group, and the zero-adjustment group only contained medium and CCK. Three 96-well plates were set for 24 h, 48 h, and 72 h. The cells were treated for the first 2 h, washed three times with sterile PBS, and then 90 μL of culture medium and 10 μL of CCK reagent were added to each well. Incubate in the incubator for 1 h, use a microplate reader to measure the OD value of each well at 450 nm and record, and the CCK value is the value obtained by subtracting the blank well from the measured value.

### 4.6. Cell Migration Assay

The wound healing assay, as well as the transwell assay, were used to assess cell migration. For the wound healing assay: cells were seeded into triplicate wells of a 6-well plate and cultured to 30–50% confluence, and then a 20 μL pipette tip was used to create artificial scratches. Cell images were collected under a microscope at 0 h, 24 h, and 48 h, and the area of the scratched area was measured by software to calculate the migration rate. For the transwell assay: 24 h after transfection, 1 × 10^5^ cells were inoculated in a 200 μL serum-free medium in the upper Transwell chamber. A total of 500 μL complete medium containing 10% FBS was added into the lower chamber as a chemoattractant. After 48 h of incubation, we used a cotton swab to remove non-invasive cells manually. Subsequently, the cells were fixed in 4% paraformaldehyde for 30 min, stained with crystal violet for 20 min, and then counted under a microscope.

### 4.7. Cell Invasion Assay

A Transwell chamber (Corning, Corning, NY, USA) with a Matrigel matrix (Corning, Corning, NY, USA) was used to measure the invasive ability of cells. Before cell plating, the Matrigel matrix was prepared with serum-free DMEM at 200 µg/mL, and 100 μL was evenly spread in each chamber. After clotting, 1 × 10^5^ serum-free cultured cells were seeded. An amount of 500 μL complete medium containing 10% FBS was added into the lower chamber as a chemoattractant. After 48 h of incubation, we used a cotton swab to remove non-invasive cells manually. Subsequently, the cells were fixed in 4% paraformaldehyde for 30 min, stained with crystal violet for 20 min, and then counted under a microscope.

### 4.8. Dual-Luciferase Reporter Assay

The HEK-293T cells were seeded in 96 wells of human embryonic kidney cells, and the density reached 50–70%. An amount of 10 µL DMEM, 0.16 µg *HOTAIR*/*CAV1* target plasmid (HanBIO, Shanghai, China), and 5 pmol *miR-203* mimics/negative control (NC) were mixed. After thorough mixing (Solution A), let this stand for 5 min at room temperature. Mix 10 µL DMEM with 0.3 µL transfection reagent (HanBIO, Shanghai, China, 0.8 mg/mL) thoroughly (Solution B), and let this stand for 5 min at room temperature. Mix solution A and solution B well and leave them at room temperature for 20 min. Add the transfection mixture to the cell culture medium and mix at 37 °C, 5% CO_2_. After 6 h of transfection, the fresh medium was changed, and 48 h after transfection, the cells were collected with a kit (Promega Dual-Luciferase, Madison, WI, USA) to measure the luminescence value of the reporter gene.

### 4.9. Western Blot

Forty-eight hours after the transfection of MDA-MB-231 cells, cell lysates were collected using 100 μL of RIPA lysate (Applygen, Beijing, China) and 10 μL of PMSF per well of a six-well plate. The protein concentration of cell lysates was measured with a BCA protein assay kit (Applygen, Beijing, China). Proteins were separated by sodium dodecyl sulfate-polyacrylamide gel electrophoresis (SDS-PAGE) and electrotransferred to nitrocellulose (PVDF) (Millipore, Boston, MA, USA) membranes for Western blotting. The membrane was blocked with 5% nonfat dry milk (BD, Franklin, LA, USA) for 2 h, and then diluted with primary antibody caveolin-1 (Affinity, Shanghai, China, 1:1000). β-actin (ZSGB-Bio, Beijing, China, 1:1000) was used as an internal reference. After overnight incubation, the membranes were washed 3 times with TBST (supplemented with 0.1% Tween 20) and incubated with HPR-labeled goat anti-rabbit IgG (ZSGB-Bio, Beijing, China, 1:1000) dilution for 2 h at room temperature. After washing with TBST, it was reacted with a luminescent solution (TransGen Biotech, Beijing, China) and developed under a gel imaging system.

### 4.10. Statistical Analysis

We statistically analyzed and calculated the mean ± standard deviation ($\bar{X}$ ± SD). The charts and experimental data analyzed were created by using GraphPad Primer 8. A student’s *t*-test was used to analyze the differences between two groups, and one-way analysis of variance (ANOVA) was used to analyze the comparison among multiple groups. A *p* < 0.05 was defined as the differences that were statistically significant. Further, ns represented no statistically significant difference between data. Image Pro Plus v.6.0 was used to analyze the wound healing and transwell assays. ImageJ software (1.8.0.112) was used to analyze the results from the Western blots.

## 5. Conclusions

**Figure 7:**
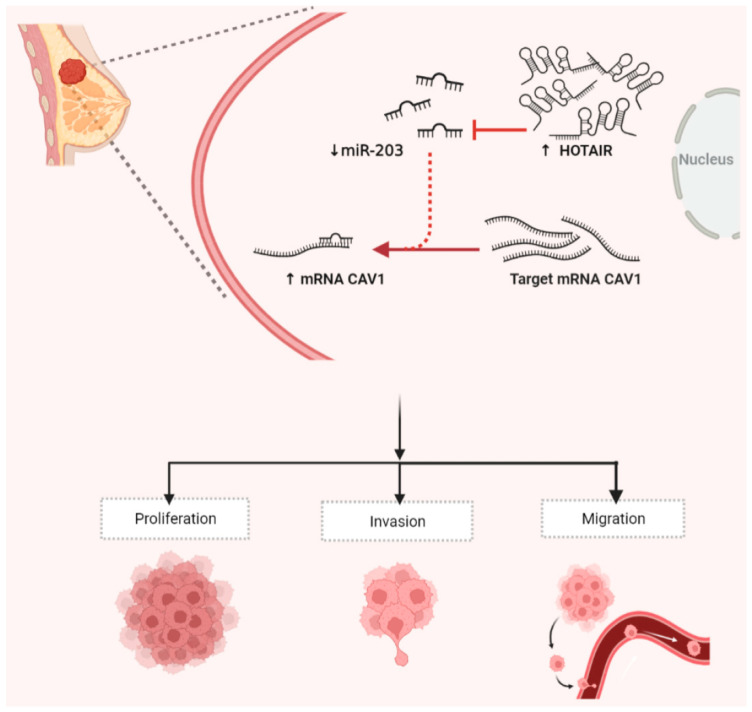
*HOTAIR*/*miR-203*/*CAV1* crosstalk influences proliferation, migration, and invasion in breast cancer cells. In breast cancer cells, the highly expressed *HOTAIR* might act as a ceRNA to bind with *miR-203*, which increases the expression of *CAV1*, thereby promoting the proliferation, invasion, and migration of breast cancer cells. The figure was constructed with BioRender (https://biorender.com/, accessed on 11 May 2022).

We successfully constructed the *HOTAIR/miR-203/CAV1* network and explored the regulatory mechanism of ceRNA-miRNA-mRNA in this network for MDA-MB-231 cells. If only the overexpression of *HOTAIR* or overexpression of *CAV1*, or the inhibition of the expression of *miR-203* is considered, the proliferation, invasion, and migration of breast cancer MDA-MB-231 cells can be significantly improved. Notably, in our H*OTAIR/miR-203/CAV1* network, *HOTAIR* competes for binding to *miR-203*, which, in turn, silences *CAV1* expression. Therefore, we can boldly propose that, once the expression of *HOTAIR* is extremely low, the resistance to *miR-203* expression is successfully reduced. Then, a large amount of *miR-203* is expressed, which, in turn, blocks the production of a large amount of *CAV1*, thereby successfully inhibiting the progression of breast cancer cells (Figure 7). As our initial validation demonstrates, knockdown of *HOTAIR* inhibited the proliferation, invasion, and migration of breast cancer cells.

Although the *HOTAIR/miR-203/CAV1* network is of great significance to the progression of breast cancer in MDA-MB-231, what other regulators are involved? The anti-tumor properties of *miR-203* and the mechanism of differential expression of related molecules in invasive and non-invasive breast cancer cells deserve further study. However, can these relevant molecules be used as targets for inhibiting breast cancer metastasis? It deserves more exploration. In the future, we will fully decipher this ceRNA/mRNA axis and provide new ideas, directions, and an experimental basis for breast cancer diagnosis, targeted therapy, and prognosis assessment.

## Data Availability

Publicly available datasets were analyzed in this study. These data can be found at: GEPIA (http://gepia.cancer-pku.cn/detail.php, accessed on 12 March 2022), UALCAN (http://ualcan.path.uab.edu/index.html, accessed on 12 March 2022), ENCORI (StarBase, https://starbase.sysu.edu.cn/index.php, accessed on 12 March 2022), DAVID (https://david.ncifcrf.gov/tools.jsp, accessed on 12 March 2022) miEAA 2.0 online (https://ccb-compute2.cs.uni-saarland.de/mieaa, accessed on 12 March 2022), GeneCards (https://www.genecards.org/, accessed on 12 March 2022) and STRING (https://cn.string-db.org/, accessed on 12 March 2022).

## Supplementary Materials

The following supporting information can be downloaded at: https://www.mdpi.com/article/10.3390/ijms231911755/s1.
