# Supplementary material for: HOTAIR/miR-203/CAV1 Crosstalk Influences Proliferation, Migration, and Invasion in the Breast Cancer Cell
Source: Int J Mol Sci. 2022 Oct 4;23(19):11755. doi: 10.3390/ijms231911755 (PMC9569926; doi:10.3390/ijms231911755)
Supplement: Supplementary file 1 [file ijms-23-11755-s001.zip › Table S3.pdf]

Table S3. The nucleotide sequence of Negative control, siRNA of Caveolin-1 and HOTAIR, and miR-203 mimics/inhibitor.

**Name**	**Sequence (5'-3')**
Negative control	UUCUCCGAACGUGUCACGUTT
	ACGUGACACGUUCGGAGAATT
Caveolin1-homo-710	GCCGUGUCUAUUCCAUCUATT
	UAGAUGGAAUAGACACGGCTT
HOTAIR-homo-536	GCCUUCCUUAUAAGCUCGUTT
	ACGAGCUUAUAAGGAAGGCTT
miR-203mimics	GUGAAAUGUUUAGGACCACUAG
	AGUGGUCCUAAACAUUUCACUU
miR-203 inhibitor	CUAGUGGUCCUAAACAUUUCAC
