# Supplementary material for: HOTAIR/miR-203/CAV1 Crosstalk Influences Proliferation, Migration, and Invasion in the Breast Cancer Cell
Source: Int J Mol Sci. 2022 Oct 4;23(19):11755. doi: 10.3390/ijms231911755 (PMC9569926; doi:10.3390/ijms231911755)
Supplement: Supplementary file 1 [file ijms-23-11755-s001.zip › Table S4.pdf]

Table S4. The information on fluorescence quantitative PCR primers.

Gene	Primer	Sequence (5'-3')
GAPDH	Forward	TCATCATCTCTGCCCCCTCT
	Reverse	AGTGATGGCATGGACTGTGG
U6	Forward	CTCGCTTCGGCAGCACA
	Reverse	AACGCTTCACGAATTTGCGT
miR-203	Forward	GGGGTGAAATGTTTAGGAC
	Reverse	CAGTGCGTGTCGTGGAGT
HOTAIR	Forward	ACATTCTGCCCTGATTTCC
	Reverse	AGTGCCTGGTGCTCTCTTAC
Caveolin-1	Forward	GCGACCCTAAACACCTCAAC
	Reverse	ATGCCGTGTCAAACTGTGTGTC
