# Supplementary material for: HOTAIR/miR-203/CAV1 Crosstalk Influences Proliferation, Migration, and Invasion in the Breast Cancer Cell
Source: Int J Mol Sci. 2022 Oct 4;23(19):11755. doi: 10.3390/ijms231911755 (PMC9569926; doi:10.3390/ijms231911755)

miRNA / precursor to category heatmap (top 100 by p-value)

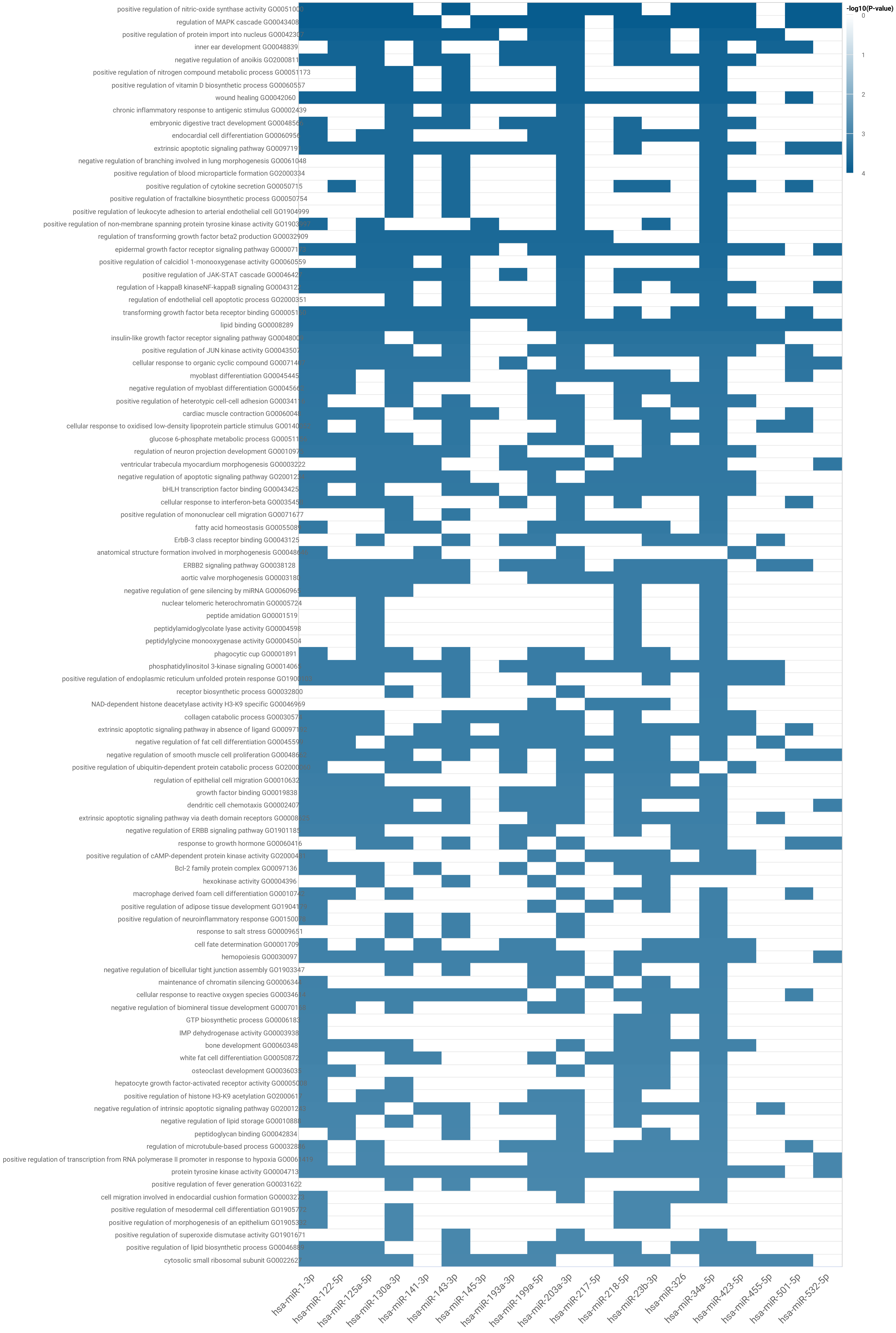

Supplement: Supplementary file 1 [file Figure S3.pdf › Figure S3.pdf]
