# Supplementary material for: HOTAIR/miR-203/CAV1 Crosstalk Influences Proliferation, Migration, and Invasion in the Breast Cancer Cell
Source: Int J Mol Sci. 2022 Oct 4;23(19):11755. doi: 10.3390/ijms231911755 (PMC9569926; doi:10.3390/ijms231911755)
Supplement: Supplementary file 1 [file Figure S4.pdf › Figure S4.pdf]

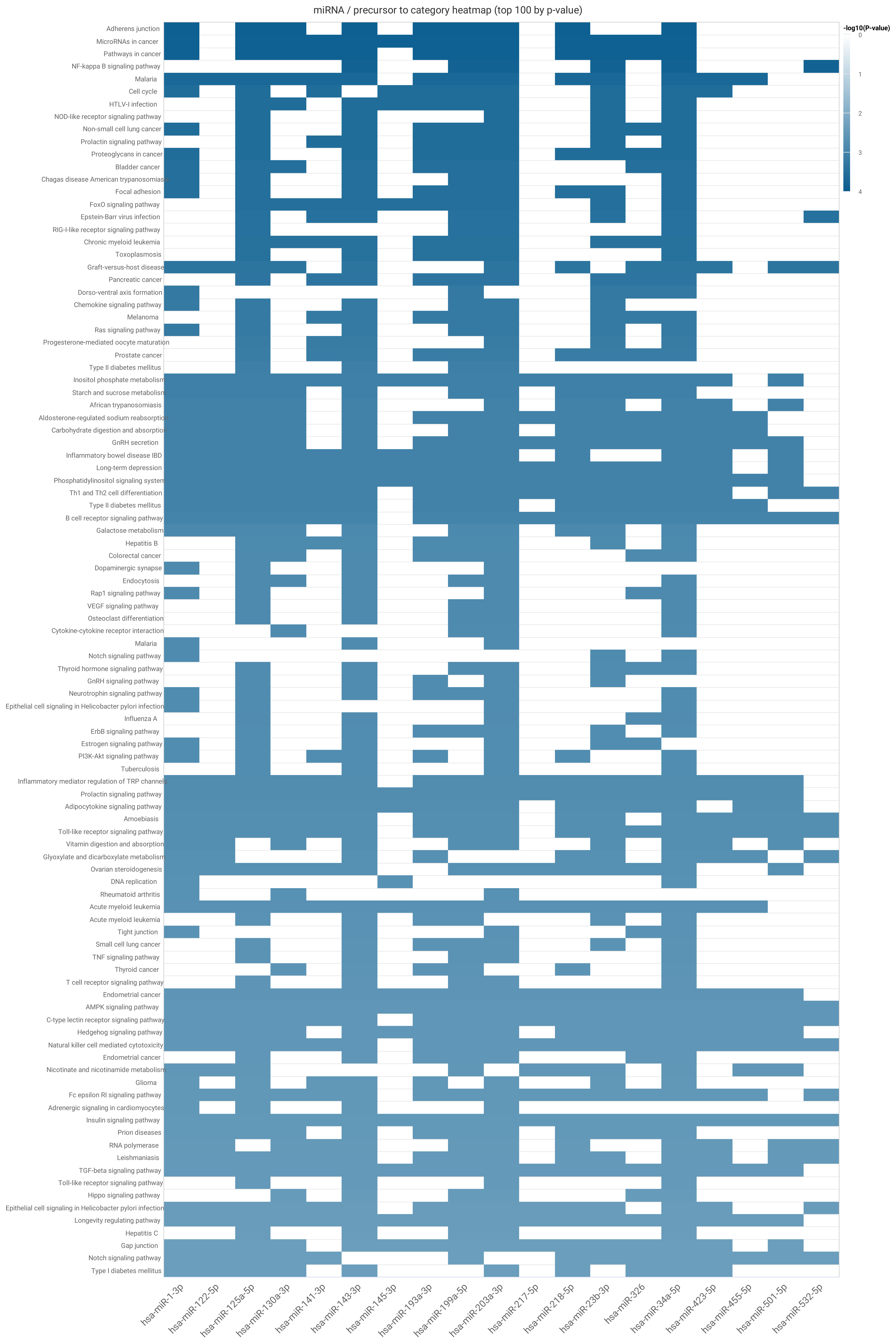

miRNA / precursor to category heatmap (top 100 by p-value)
-log10(P-value)
0
1
2
3
4
Adherens junction
MicroRNAs in cancer
Pathways in cancer
NF-kappa B signaling pathway
Malaria
Cell cycle
HTLV-I infection
NOD-like receptor signaling pathway
Non-small cell lung cancer
Prolactin signaling pathway
Proteoglycans in cancer
Bladder cancer
Chagas disease American trypanosomiasis
Focal adhesion
FoxO signaling pathway
Epstein-Barr virus infection
RIG-I-like receptor signaling pathway
Chronic myeloid leukemia
Toxoplasmosis
Graft-versus-host disease
Pancreatic cancer
Dorso-ventral axis formation
Chemokine signaling pathway
Melanoma
Ras signaling pathway
Progesterone-mediated oocyte maturation
Prostate cancer
Type II diabetes mellitus
Inositol phosphate metabolism
Starch and sucrose metabolism
African trypanosomiasis
Aldosterone-regulated sodium reabsorption
Carbohydrate digestion and absorption
GnRH secretion
Inflammatory bowel disease IBD
Long-term depression
Phosphatidylinositol signaling system
Th1 and Th2 cell differentiation
Type II diabetes mellitus
B cell receptor signaling pathway
Galactose metabolism
Hepatitis B
Colorectal cancer
Dopaminergic synapse
Endocytosis
Rap1 signaling pathway
VEGF signaling pathway
Osteoclast differentiation
Cytokine-cytokine receptor interaction
Malaria
Notch signaling pathway
Thyroid hormone signaling pathway
GnRH signaling pathway
Neurotrophin signaling pathway
Epithelial cell signaling in Helicobacter pylori infection
Influenza A
ErbB signaling pathway
Estrogen signaling pathway
PI3K-Akt signaling pathway
Tuberculosis
Inflammatory mediator regulation of TRP channels
Prolactin signaling pathway
Adipocytokine signaling pathway
Amoebiasis
Toll-like receptor signaling pathway
Vitamin digestion and absorption
Glyoxylate and dicarboxylate metabolism
Ovarian steroidogenesis
DNA replication
Rheumatoid arthritis
Acute myeloid leukemia
Acute myeloid leukemia
Tight junction
Small cell lung cancer
TNF signaling pathway
Thyroid cancer
T cell receptor signaling pathway
Endometrial cancer
AMPK signaling pathway
C-type lectin receptor signaling pathway
Hedgehog signaling pathway
Natural killer cell mediated cytotoxicity
Endometrial cancer
Nicotinate and nicotinamide metabolism
Glioma
Fc epsilon RI signaling pathway
Adrenergic signaling in cardiomyocytes
Insulin signaling pathway
Prion diseases
RNA polymerase
Leishmaniasis
TGF-beta signaling pathway
Toll-like receptor signaling pathway
Hippo signaling pathway
Epithelial cell signaling in Helicobacter pylori infection
Longevity regulating pathway
Hepatitis C
Gap junction
Notch signaling pathway
Type I diabetes mellitus
hsa-miR-1-3p
hsa-miR-122-5p
hsa-miR-125a-5p
hsa-miR-130a-3p
hsa-miR-141-3p
hsa-miR-143-3p
hsa-miR-145-3p
hsa-miR-193a-3p
hsa-miR-199a-5p
hsa-miR-203a-3p
hsa-miR-217-5p
hsa-miR-218-5p
hsa-miR-23b-3p
hsa-miR-326
hsa-miR-34a-5p
hsa-miR-423-5p
hsa-miR-455-5p
hsa-miR-501-5p
hsa-miR-532-5p
